# Long-term mortality among older adults with burn injury: a population-based study in Australia

**DOI:** 10.2471/BLT.14.149146

**Published:** 2015-04-20

**Authors:** Janine M Duke, James H Boyd, Suzanne Rea, Sean M Randall, Fiona M Wood

**Affiliations:** a Burn Injury Research Unit, School of Surgery, Faculty of Medicine, Dentistry and Health Sciences, University of Western Australia, M318 35 Stirling Highway, Crawley, 6009, Perth, Western Australia, Australia.; b Centre for Data Linkage, Curtin University, Perth, Australia.; c Burns Service of Western Australia, Royal Perth Hospital and Princess Margaret Hospital, Perth, Australia.

## Abstract

**Objective:**

To assess if burn injury in older adults is associated with changes in long-term all-cause mortality and to estimate the increased risk of death attributable to burn injury.

**Methods:**

We conducted a population-based matched longitudinal study – based on administrative data from Western Australia’s hospital morbidity data system and death register. A cohort of 6014 individuals who were aged at least 45 years when hospitalized for a first burn injury in 1980–2012 was identified. A non-injury comparison cohort, randomly selected from Western Australia’s electoral roll (*n* = 25 759), was matched to the patients. We used Kaplan–Meier plots and Cox proportional hazards regression to analyse the data and generated mortality rate ratios and attributable risk percentages.

**Findings:**

For those hospitalized with burns, 180 (3%) died in hospital and 2498 (42%) died after discharge. Individuals with burn injury had a 1.4-fold greater mortality rate than those with no injury (95% confidence interval, CI: 1.3–1.5). In this cohort, the long-term mortality attributable to burn injury was 29%. Mortality risk was increased by both severe and minor burns, with adjusted mortality rate ratios of 1.3 (95% CI: 1.1–1.9) and 2.1 (95% CI: 1.9–2.3), respectively.

**Conclusion:**

Burn injury is associated with increased long-term mortality. In our study population, sole reliance on data on in-hospital deaths would lead to an underestimate of the true mortality burden associated with burn injury.

## Résumé

**Objectif:**

Évaluer si les brûlures, chez les adultes âgés, sont associées à un changement de la mortalité à long terme toutes causes confondues et estimer la majoration du risque de décès attribuable aux brûlures.

**Méthodes:**

Nous avons réalisé une étude longitudinale appariée en population, à partir de données administratives extraites des registres de décès et du système de données sur la morbidité des hôpitaux d'Australie occidentale. Une cohorte de 6 014 individus, âgés d'au moins 45 ans au moment de leur première hospitalisation pour brûlures, sur la période de 1980 à 2012, a été identifiée. Une cohorte comparative d'individus n'ayant pas souffert de brûlures a été aléatoirement sélectionnée dans les listes électorales d'Australie occidentale (n = 25 759) pour appariement avec la cohorte des patients victimes de brûlures. Nous avons utilisé des courbes de Kaplan-Meier et une régression des risques proportionnels de Cox pour analyser les données et obtenir les ratios des taux de mortalité et les pourcentages de risque attribuable.

**Résultats:**

Dans la cohorte des personnes hospitalisées pour brûlures, 180 personnes (3 %) sont décédées à l'hôpital et 2 498 personnes (42 %) sont décédées après leur sortie de l'hôpital. Nous avons trouvé un taux de mortalité 1,4 fois supérieur pour les victimes de brûlures comparativement aux individus non victimes de brûlures (intervalle de confiance de 95 % : 1,3-1,5). Dans cette cohorte, la mortalité à long terme attribuable aux brûlures a été de 29 %. Le risque de mortalité a été majoré à la fois en cas de brûlures graves et de brûlures mineures, avec des ratios de taux de mortalité ajustés de 1,3 (IC de 95 % : 1,1-1,9) et 2,1 (IC de 95 % : 1,9-2,3) respectivement.

**Conclusion:**

Les brûlures sont associées à une majoration de la mortalité à long terme. Dans la population étudiée, l'utilisation exclusive des données relatives aux décès survenus dans les hôpitaux aurait entraîné une sous-estimation de la vraie charge de mortalité associée aux brûlures.

## Resumen

**Objetivo:**

Evaluar si las quemaduras en adultos de edad avanzada están asociadas con los cambios en la mortalidad por todas las causas a largo plazo y estimar el aumento del riesgo de muerte atribuible a las quemaduras.

**Métodos:**

Se llevó a cabo un estudio poblacional longitudinal emparejado sobre la base de datos administrativos proporcionados por el sistema de datos de morbilidad y el registro de defunciones de los hospitales de Australia Occidental. Se identificó una cohorte de 6014 individuos que tenían como mínimo 45 años cuando fueron hospitalizados por una primera quemadura entre 1980 y 2012. Se comparó una cohorte de personas que no habían sufrido quemaduras seleccionadas aleatoriamente del censo electoral de Australia Occidental (n=25 759) con los pacientes. Se utilizaron el método de Kaplan-Meier y la regresión de Cox de riesgos proporcionales para analizar los datos, las razones de tasas de mortalidad generados y los porcentajes de riesgo atribuible.

**Resultados:**

De todos los hospitalizados con quemaduras, 180 (el 3%) murieron en el hospital y 2498 (el 42%) murieron después de haber sido dados de alta. Los individuos con quemaduras tenían una tasa de mortalidad 1,4 veces mayor a la de los individuos sin quemaduras (intervalo de confianza, IC, del 95%: 1,3–1,5). En esta cohorte, la mortalidad a largo plazo atribuible a las quemaduras resultó ser del 29%. El riesgo de mortalidad se incrementó tanto por quemaduras graves como por quemaduras leves, con unas razones de tasas de mortalidad ajustadas de 1,3 (IC del 95%: 1,1-1,9) y 2,1 (IC del 95%: 1,9-2,3), respectivamente.

**Conclusión:**

Las quemaduras están asociadas al aumento de la mortalidad a largo plazo. En nuestra población de estudio, confiar exclusivamente en los datos relativos a las muertes en hospitales llevaría a una subestimación de la verdadera carga de mortalidad asociada con las quemaduras.

## ملخص

**الغرض:**

تقييم ما إذا كانت إصابات الحروق في البالغين الأكبر سنًا مرتبطة بتغيرات في معدَّل الوفيات لجميع الأسباب على المدى الطويل وتقدير الاختطار الزائد للموت الذي يعزى إلى الإصابات الناتجة عن الحروق.

**الطريقة:**

أجرينا دراسة طولانية مطابقة قائمة على السكان – تستند إلى البيانات الإدارية المأخوذة من نظام بيانات المراضة وسجل الوفيات الخاص بمستشفيات ولاية أستراليا الغربية. تم تحديد مجموعة من **  **6014 من الأفراد البالغ أعمارهم **  **45 سنة على الأقل عند إدخالهم إلى المستشفى للعلاج من إصابات الحروق للمرة الأولى من عام **  **1980 إلى عام **  **2012. تمت مطابقة دراسة حشدية مقارنة لغير الإصابات والتي تم اختيارها عشوائيًا من السجل الانتخابي في ولاية أستراليا الغربية ( *العدد ***  **= **  **25 759) مع المرضى. ولقد استخدمنا مخططات كابلان–ماير ونماذج تحوف المخاطر التناسبية لكوكس لتحليل البيانات ونسب معدلات الوفيات المستحدثة والنسب المئوية للأخطار التي تعزى إليها.

**النتائج:**

بالنسبة للأشخاص الذين تم إدخالهم إلى المستشفى للعلاج من الإصابة بالحروق، توفى 180 (بنسبة 3‏ %) شخصًا منهم في المستشفى وتوفى 2498 (بنسبة ‏‏42‏ %) شخصًا بعد خروجهم من المستشفى. وكان معدل وفيات الأفراد الذين يعانون من إصابات الحروق أكبر بـ1.4 مرة من هؤلاء الأفراد الذين لا يعانون من أي إصابة (بنسبة أرجحية مقدارها ‏95‏ %: 1.3–1.5). في هذه المجموعة، كانت نسبة الوفيات طويلة الأمد التي تعزى إلى إصابات الحروق قد بلغت 29‏ %. ولقد زاد اختطار الوفيات جراء الإصابة بكلاً من الحروق الوخيمة والخفيفة مع نسب معدلات الوفيات المصححة من 1.3 (بنسبة أرجحية مقدارها ‏95‏‏ %: 1.1–1.9) و2.1 (بنسبة أرجحية مقدارها 95‏‏ %: 1.9–2.3)، على التوالي.

**الاستنتاج:**

ترتبط إصابات الحروق بزيادة نسبة الوفيات طويلة الأمد. في دراسة السكان التي أجريناها، وجدنا أن الاكتفاء بالاتكال على البيانات الصادرة عن حالات الوفيات في داخل المستشفى قد يؤدي إلى التقليل من تقدير عبء معدلات الوفيات الحقيقية المرتبطة بإصابات الحروق.

## 摘要

**目的:**

旨在评估老年人群的烧伤情况是否与各种原因造成的长期死亡率的变化有关，并估计因烧伤而增加的死亡风险。

**方法:**

我们开展了一项以人群为基础的配对式纵向研究——基于从西澳大利亚的医院发病率数据系统和死亡登记处采集的管理数据。现已确定有 6014 个人 1980 – 2012 年期间因首次烧伤而住院且年龄至少为 45 岁。从西澳大利亚的选民名册 (*n* = 25 759) 中随机选择的非受伤对比人群与患者相匹配。我们采用了 Kaplan–Meier 法和 Cox 比例风险回归法来分析数据和产生的死亡比率以及归属原因的风险百分比。

**结果:**

在那些因烧伤而住院的人群中，180 (3%) 名在医院死亡，2498 (42%) 名在出院后死亡。烧伤个人的死亡率比非受伤人群的死亡率高 1.4 倍（95% 置信区间，CI：1.3 - 1.5）。在该类人群中，因烧伤造成的长期死亡率为 29%。死亡风险因严重烧伤和轻微烧伤而增加，调整后的死亡比率分别为 1.3 (95% CI:1.1 - 1.9) 和 2.1 (95% CI:1.9 - 2.3)。

**结论:**

烧伤与长期死亡率的增加有关。在我们的研究人群中，单纯依靠院内死亡数据将会导致在因烧伤造成的死亡率方面低估真正的量。

## Резюме

**Цель:**

Оценка вероятной связи ожоговых травм у пациентов преклонного возраста с изменениями показателей общей смертности в отдаленном периоде, а также оценка повышенного риска смертельного исхода в результате ожоговой травмы.

**Методы:**

Проведено популяционное сравнительное продольное исследование на основании административных данных о заболеваемости и книг регистрации актов смерти больницы Западной Австралии. Была определена группа из 6014 человек, в которую входили лица старше 45 лет, госпитализированные с первой ожоговой травмой за период с 1980 по 2012 год. Контрольная группа без травм, выбранная произвольно из списка избирателей Западной Австралии (*n* = 25 759), сравнивалась с данными пациентов. Для анализа данных, полученных коэффициентов смертности и процентов добавочного риска использовались диаграммы Каплана-Мейера и регрессивный анализ пропорциональных рисков Кокса.

**Результаты:**

Из пациентов, госпитализированных с ожогами, 180 человек (3%) умерли в больнице и 2498 человек (42%) умерли после выписки из больницы. Лица с ожоговыми травмами имели в 1,4 раза больший коэффициент смертности, чем их соотечественники, не имевшие травм (доверительный интервал 95%, CI: 1,3–1,5). В этой группе смертность в отдаленном периоде, приписываемая ожоговым травмам, составила 29%. Риск смертности возрастал как при тяжелых, так и при легких ожогах, коэффициент смертности равнялся 1,3 (95% CI: 1,1–1,9) и 2,1 (95% CI: 1,9–2,3) соответственно.

**Вывод:**

Ожоговые травмы связаны с ростом показателей смертности в отдаленном периоде. Что касается популяции нашего исследования, то использование исключительно данных больничной смертности приводит к недооценке истинного коэффициента смертности, связанного с ожоговыми травмами.****

## Introduction

Burn injury is an important cause of morbidity and mortality worldwide, particularly among older adults.^1^^–^^6^ In high-income countries, where older adults form an increasingly large proportion of the population, the incidence of burn injury is likely to increase. ^7^ Compared with burn injuries in younger individuals, burn injuries in older adults cause increased physical impairment, reductions in the quality of life, loss of independence and increased mortality.^8^^,^^9^

Improvements in the understanding of the pathophysiology of burns over the past decades have led to advances in medical and surgical treatment. The probability that an older adult is discharged alive after admission for an acute burn appears to be increasing.^2^^,^^3^^,^^10^ However, compared with younger people, older adults with burn injury are still more likely to die in the year^8^^,^^11^ or two years^12^ following their discharge and often have to be readmitted because of pre-existing comorbidities and ongoing chronic illness.^8^^,^^9^^,^^12^

Using data on adults who have been hospitalized for burns in Western Australia, we investigate long-term mortality and the proportion of mortality attributable to the original burn injury.

## Methods

Our study formed part of the Western Australian population-based burn injury project – a retrospective cohort investigation that uses administrative data from the Western Australian data linkage system. Administrative health data from several core data sets – including Western Australia’s hospital morbidity data system and death register – are linked for the entire population of Western Australia.^13^ The project protocol was approved by the human research ethics committees of the University of Western Australia and the Western Australian department of health.

Staff at the Western Australian data linkage system provided a de-identified extraction of hospital morbidity records for all individuals who were aged at least 45 years when admitted to a hospital in Western Australia with a first burn injury between 1 January 1980 and 30 June 2012. Other than the unique identifying numbers assigned by staff at the Western Australian data linkage system, personal identifiers were removed from the data. We used the International Classification of Diseases and Related Health Problems (ICD9) CM 940–949 or (ICD10) AM T20–T31 codes to identify burn injuries. A first burn injury was defined as the first hospital admission in a patient’s medical record in which a burn injury was given as the principal diagnosis or an additional diagnosis. A population-based comparison cohort was randomly selected from Western Australia’s electoral roll. Any person with an injury hospitalization during the study period was excluded from this cohort by staff at the Western Australian data linkage system. The resultant comparison cohort was frequency matched on birth year and sex of each burn injury case – with four controls to each case – for each year from 1980 to 2012.

Data from Western Australia’s hospital morbidity data system and death register were linked to the burn and non-injured cohorts for the period 1980–2012. Hospital admissions’ data included principal and additional diagnoses, external cause of injury, age, sex, Aboriginal status, date of admission, date of discharge or other separation, mode of separation, percentage of total body surface area burned and burn depth. The data also included geocoded place of residence (census collectors’ district or postcode), geocoded indices of geographical remoteness^14^ and social disadvantage.^15^ Geographical remoteness was classified into five categories: major cities, inner regional, outer regional, remote and very remote. The social disadvantage index was reclassified into quintiles. The mortality data included date of death and cause of death, classified using ICD9-CM and ICD10-AM disease and external cause codes.

Individuals aged 45–54, 55–64 and at least 65 years were categorized as middle-aged, young-old and elderly, respectively. Individuals listed as Aboriginal or Torres Strait Islander on any admission record were categorized as Aboriginal. Supplementary codes ICD9-CM 948 or ICD10-AM T31, when available, were used to classify the patients into those with minor burns (less than 20% of total body surface area burned) and those with severe burns. Comorbidity was assessed, with a five-year look-back period, using the Charlson comorbidity index^16^ and the principal and additional diagnoses included in the hospital morbidity records.^17^ The final discharge date for the burn patient was used as the starting point for the follow-up periods for both the burn injury and control cohort. 

Categorical and non-parametric continuous variables were compared using *χ^2^* and Kruskal–Wallis tests, respectively. A *P*-value of 0.05 or lower was considered statistically significant. Kaplan–Meier plots of survival estimates were generated and log rank tests were used to compare the survival distributions of the burn and non-injury cohorts. We compared burn versus non-injury and burn severity (minor burns, severe burns or burns with no record of the percentage of total body surface area affected) versus non-injury. Cox proportional hazard regression was used to estimate the effects of burn injury on long-term survival while adjusting for year of admission, age, sex, Aboriginal status, comorbidity score, social disadvantage, and geographical remoteness. The hazard ratios estimated from the Cox proportional hazards model were used as measures of mortality rate ratios. Preliminary analyses revealed no evidence of non-proportionality.^18^ Attributable risk percentage – used to estimate the proportion of long-term mortality for which burn injury was an attributable cause – was calculated as 100 × (adjusted mortality rate ratio–1)/(adjusted mortality rate ratio).^19^

The percentage of deaths in the burn cohort that were attributable to burn injury was estimated after adjusting for potential confounders. All statistical analyses were performed using Stata version 12 (StataCorp. LP, College Station, United States of America).

## Results

During the study period, 6014 individuals aged 45 years or older were hospitalized in Western Australia for a first burn injury. Although 240 (4%) were recorded as having severe burns and 3248 (54%) as having minor burns, the percentage of the total body surface area affected by burns was not recorded for the remaining 2526 (42%). Overall, 1143 (19%) of patients had full-thickness burns, while 2225 (37%) had partial-thickness burns, 1082 (18%) had erythema and 1744 (29%) had burns of unspecified depth. Patients may have had multiple burns sites and depths recorded. In total, 4992 (83%) of patients were discharged to their home, 722 (12%) were transferred to acute care hospitals, 120 (2%) were transferred to facilities for elderly people and 180 (3%) died in hospital. Only 577 (10%) had additional non-burn injuries, most of which were open wounds or superficial injuries. Mortality among individuals with and without additional injury were similar (43% versus 41%; *P* = 0.356). Additional injuries in the burn cohort were therefore ignored in our subsequent analyses.

Our non-injury cohort comprised 25 759 individuals. Table 1 summarizes the baseline sociodemographic characteristics and comorbidity scores for both study cohorts. Compared with the non-injury cohort, the burn cohort had significantly higher proportions of Aboriginal people, people who were socially disadvantaged and people living outside major cities. The adults in the burn cohort were also significantly more likely to have pre-existing comorbidity than those in the non-injury cohort.

**Table 1:** Long-term mortality following burn injury: baseline cohort characteristics, Western Australia, 1980–2012

Characteristic	Burn cohort No. (%)	Non-injury cohort No. (%)	*P*
Total	6014 (100)	25 759 (100)	
Male	3756 (62)	15 970 (62)	0.787
**Age, years**			0.374
45–54	2279 (38)	10 046 (39)	
55–64	1479 (24)	6182 (24)	
≥ 65	2256 (38)	9531 (37)	
**Aboriginal status**			< 0.001
No	5351 (89)	25 507 (99)	
Yes	663 (11)	252 (1)	
**Social disadvantage quintile**^a^			< 0.001
1	1293 (22)	3423 (14)	
2	1792 (30)	5239 (22)	
3	1209 (20)	4309 (18)	
4	773 (13)	4337 (18)	
5	880 (15)	6790 (28)	
**Remoteness**^b^			< 0.001
Major city	3329 (55)	18 006 (75)	
Inner regional	716 (12)	2758 (11)	
Outer regional	903 (15)	2095 (9)	
Remote	495 (8)	766 (3)	
Very remote	496 (8)	473 (2)	
**Comorbidity score**^c^			< 0.001
0	3917 (65)	22 289 (87)	
1	403 (7)	733 (3)	
2	348 (6)	744 (3)	
3	1346 (22)	1993 (8)	

^a^ Quintiles of socioeconomic disadvantage^15^ based on the geocoded place of residence. Quintiles 1 and 5 represent the most and least disadvantaged, respectively. Complete geocoded data for 5947 (99%) burn cohort and 24 098 (94%) non-injured cohort.

^b^ Geographical remoteness^14^ based on the geocoded place of residence. Complete geocoded data for 5939 (99%) burn cohort and 24 098 (94%) non-injured cohort.

^c^ Based on the Charlson comorbidity index^16^ and a five-year look-back period.

**Fig. 1:**
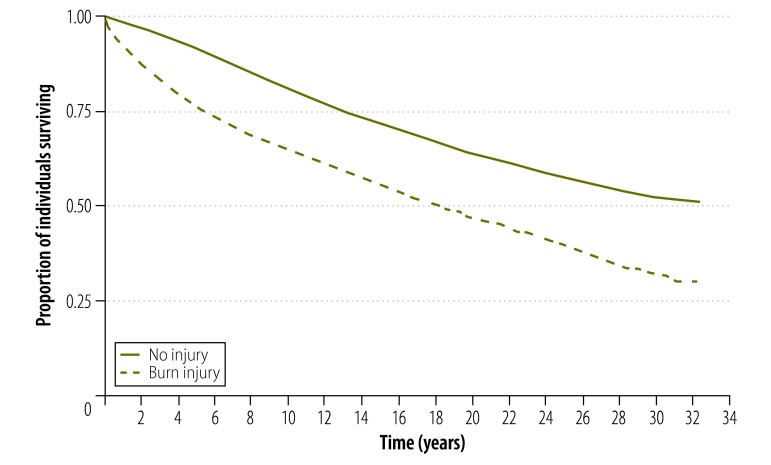
Survival of all burn patients after hospital discharge and matched non-injured controls, Western Australia, 1980–2012

Note: The plots show Kaplan–Meier estimates of the survivor function

**Fig. 2:**
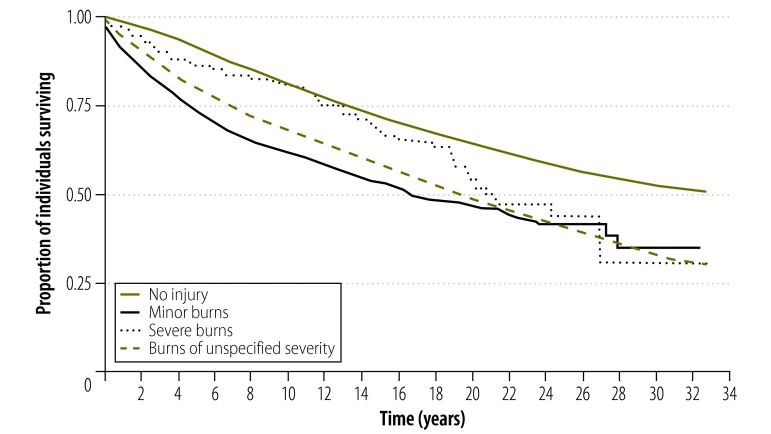
Survival of all burn patients by burn severity after discharge and matched non-injured controls, Western Australia, 1980–2012

Note: The plots show Kaplan–Meier estimates of the survivor function. Patients with minor burns had less than 20% of their total body surface area affected.

Over the study period, 2498 (42%) of the adults in the burn cohort and 7018 (27%) of those in the matched non-injury cohort died. Unadjusted Kaplan–Meier survival plots for the overall burn cohort versus the non-injury cohort (Fig. 1), and for the three separate categories for burn severity versus non-injury (Fig. 2), all showed higher survival estimates for those who had not suffered burns. Log rank tests – in which the unadjusted equality of survivorship between the non-injury cohort and the total burn cohort (*P* < 0.001) or each of the three categories for burn severity (*P* < 0.001) was assessed – all indicated that there were excess deaths in the burn cohort.

Compared with the adults in the non-injury cohort who died during the study period, the adults in the burn cohort who died were younger at the time of death – with a median age of 76 years (interquartile range: IQR: 67–85) versus 82 years (IQR: 73–88; *P* < 0.001).

Over the 33-year study period, the adults in the burn cohort were followed up for 0.01–32.5 years (median: 9; IQR: 3–16) to give a total of 59 882 person-years while those in the non-injury cohort were followed up for 0.01–32.5 years (median: 13; IQR: 6–23) to give a total of 488 443 person-years. Over the study period, the burn and non-injury cohorts had all-cause mortality rates of 419.4 and 143.7 deaths per 10 000 person-years, respectively – giving an unadjusted mortality rate ratio for burn injury of 2.9 (95% confidence interval, CI: 2.7–3.0). After adjustment for year of admission, age, sex, Aboriginal status, social disadvantage, remoteness and pre-existing comorbidity, the overall long-term adjusted mortality rate ratio for burn injury was 1.4 (95% CI: 1.3–1.5). Mortality attributable to burns was 29% (Table 2).

**Table 2:** Mortality rate ratios and attributable risk percentages for individuals with burn injuries, Western Australia, 1980–2012

Group compared with non-injury cohort	No. of deaths	MRR (95% CI)		AR, %
		Unadjusted	Adjusted	
Entire burn cohort	2498	2.9 (2.7–3.0)	1.4 (1.3–1.5)^a^	29^a^
Adults with minor burn injury	1070	3.4 (3.2–3.7)	2.1 (1.9–2.3)^a^	52^a^
Adults with severe burn injury	58	1.1 (0.8–1.4)	1.3 (1.1–1.9)^a^	23^a^
Adults with burn injury of unspecified severity	1370	2.6 (2.5–2.8)	1.4 (1.3–1.5)^a^	29^a^
Men with burn injury	1429	2.8 (2.7–3.0)	1.3 (1.2–1.4)^b^	23^b^
Women with burn injury	1069	2.1 (2.0–2.3)	1.6 (1.5–1.7)^b^	38^b^
Adults with burn injury aged 45–54 years when hospitalized	472	2.3 (2.1–2.6)	1.6 (1.4–1.8)^c^	38^c^
Adults with burn injury aged 55–64 years when hospitalized	584	1.9 (1.7–2.1)	1.5 (1.4–1.7)^c^	33^c^
Adults with burn injury aged ≥ 65 years when hospitalized	1442	1.6 (1.5–1.7)	1.3 (1.2–1.4)^c^	23^c^

AR: attributable risk; CI: confidence interval; MRR: mortality rate ratio.

^a^ Adjusted for age group when hospitalized, sex, Aboriginal status, social disadvantage, geographical remoteness, index year and comorbidity score.

^b^ Adjusted for age group when hospitalized, Aboriginal status, social disadvantage, geographical remoteness, index year and comorbidity score.

^c^ Adjusted for sex, Aboriginal status, social disadvantage, geographical remoteness, index year and comorbidity score.

The all-cause mortality rate among females was 538.7 deaths per 10 000 person-years in the burn cohort and 253.1 deaths per 10 000 person-years in the non-injury cohort, giving an unadjusted mortality rate ratio for burn injury of 2.1 (95% CI: 2.0–2.3). The adjusted mortality rate ratio for females was 1.6 (95% CI: 1.5–1.7). Among males, the all-cause mortality rate in the burn cohort was also higher than that in the non-injury cohort – 359.9 versus 126.0 deaths per 10 000 person-years – giving an unadjusted mortality rate ratio of 2.8 (95% CI: 2.7–3.0). The adjusted mortality rate ratio for males was 1.3 (95% CI: 1.2–1.4).

The results of Cox regression models for subgroups of the burn cohort, classified by burn severity and age, are presented in Table 2. In these models, after adjustment for potential confounders, all of the subgroups of the burn cohort had a higher risk of death than the non-injury cohort. Individuals aged at least 65 years when they experienced the burn injury had a lower attributable risk (23%), than individuals aged 55–64 years (33%) and individuals aged 45–54 years (38%).

## Discussion

Current estimates of the mortality related to burns have usually been based on deaths in hospital or within a few weeks of discharge.^4^^,^^12^^,^^20^^–^^23^ In this study, however, follow-up lasted for a median of 9 years.

Up to the end of follow-up – and after adjusting for known potential confounders – our burn cohort was found to have 1.4-fold higher all-cause mortality than the non-injury cohort. The excess long-term mortality attributable to burn injury was 725 of the 2498 total deaths recorded in the burn cohort. These 725 deaths after discharge represent 12% of those hospitalized for burn injury while only 3% died in hospital after the index admission. These results, which indicate that the long-term mortality from burns is much higher than indicated by in-hospital mortality data, challenge the definition of fatal outcomes used in most assessments of burn injuries.

Compared with the non-injury cohort, the subgroups of the burn cohort with severe burns, minor burns or burns of unspecified severity each showed significantly increased risk of all-cause mortality during the study follow-up. We failed to demonstrate a positive relationship between burn severity and mortality because, within the burn cohort, we found the mortality to be highest for those with minor burns. Explanations for this phenomenon may be that people with the most severe burns died during the index hospital admission and/or the survivors of severe burns included in the analyses represented a relatively more robust patient population.

While burn injury principally affects the skin, it is associated with depression of humoral and cell-mediated immunity,^24^^,^^25^ sustained high levels of oxidative stress^26^ and prolonged elevation of hypermetabolic and stress hormones.^27^^,^^28^ In children, metabolic and inflammatory changes have been found to last for at least three years after severe burns.^29^ Such changes have the potential to induce a range of health conditions including insulin resistance,^30^ increases in the risk of fracture,^31^ sepsis and infections,^28^^,^^32^ enlargement of the liver,^33^^,^^34^ cardiac stress and dysfunction^28^^,^^35^ and hormonal abnormalities.^30^^,^^33^ Recent research has also shown elevated cancer incidence after burn injury, particularly among female burn survivors.^36^^,^^37^ Systemic responses are induced by both minor and moderate burns^38^^,^^39^ and may have contributed to the increased long-term mortality found, for both minor and severe burns, in this study. It has also been reported that patients who survive admission to an intensive care unit have poorer survival than the general population – perhaps indicating that any episode of critical illness or treatment for such illness can shorten life expectancy.^40^

Our analysis by age group indicated that the mortality rate ratios – and consequent attributable risk percentages – for burn injury decreased slightly with increasing age. Although the proportion of deaths attributable to burn injury was smaller for the elderly individuals than the middle-aged or young-old individuals, the absolute number of deaths was greatest among the elderly individuals.

Some of the consequences of burn injury appear to be experienced long after the initial period of recovery. Minor burns in elderly people tend to lead to longer hospital stays and higher in-hospital mortality when compared with younger adults.^4^^,^^21^^,^^41^ Among elderly people, burn injuries may worsen pre-existing health conditions, hamper good nutrition, reduce mobility and independence^9^^,^^42^ and prevent them from regaining their previous state of health.^12^ An ageing population is characterized by an increasing prevalence of frailty.^43^

The strengths of our study are the inclusion of a non-injured comparison group and a follow-up period designed to reveal the long-term mortality risk associated with burns among older adults. Previous single-centre studies have lacked a control group^44^ or followed patients for no more than two to three years.^12^^,^^29^ Long-term mortality of injury has been investigated previously, but with few burn cases.^45^ We assumed that, after adjustment for confounding, the excess in mortality in the burn cohort was predominantly associated with burn injuries.^19^Our use of linked health administrative data enabled both the identification of a cohort of adults hospitalized for a first burn injury and the estimation of the pre-existing comorbidities in that cohort. We included indices of social disadvantage, geographical remoteness and access to services.

As a consequence of incomplete data for the percentage of the total body surface area affected for many people with burn injury, we were unable to show the effects of burn severity on long-term mortality. A non-injured control cohort was used to examine the potential systemic health impacts of trauma caused specifically by burn injury sufficiently serious to require hospitalization. Future research will examine potential differences between burn and non-burn injury and long-term health impacts.

The fact that the Western Australia hospital morbidity data system is assessed continually for both quality and accuracy strengthens our findings.^46^ Our main finding is expected to be generalizable to other populations with similar demographic characteristics and comparable health-care systems to those of Australia.

In conclusion, our findings that the long-term all-cause mortality was increased in the burn injury cohort suggest that any estimate of the mortality burden from burn injury based on in-hospital deaths alone will underestimate the true burden. These findings have implications for the clinical management of burn injury and should encourage the development of programmes for both long-term support for people with burn injury and the prevention of such injury.
